# In vivo multi spectral colonoscopy in mice

**DOI:** 10.1038/s41598-022-12794-1

**Published:** 2022-05-24

**Authors:** Martin Hohmann, Ingo Ganzleben, Alexander Grünberg, Jean Gonzales-Menezes, Florian Klämpfl, Benjamin Lengenfelder, Eva Liebing, Christina Heichler, Clemens Neufert, Christoph Becker, Markus F. Neurath, Maximilian J. Waldner, Michael Schmidt

**Affiliations:** 1 Institute of Photonic Technologies (LPT), Friedrich-Alexander-Universität Erlangen-Nürnberg (FAU), Konrad-Zuse-Straße 3/5, 91052 Erlangen, Germany; 2 Erlangen Graduate School in Advanced Optical Technologies (SAOT), Paul-Gordon-Straße 6, 91052 Erlangen, Germany; 3 Department of Medicine 1, University Hospital, Friedrich-Alexander-Universität, Erlangen-Nürnberg (FAU), Ulmenweg 18, 91054 Erlangen, Germany

**Keywords:** Colorectal cancer, Colonoscopy, Imaging and sensing, Optical spectroscopy, Statistics, Optics and photonics

## Abstract

Multi- and hyperspectral endoscopy are possibilities to improve the endoscopic detection of neoplastic lesions in the colon and rectum during colonoscopy. However, most studies in this context are performed on histological samples/biopsies or ex vivo. This leads to the question if previous results can be transferred to an in vivo setting. Therefore, the current study evaluated the usefulness of multispectral endoscopy in identifying neoplastic lesions in the colon. The data set consists of 25 mice with colonic neoplastic lesions and the data analysis is performed by machine learning. Another question addressed was whether adding additional spatial features based on Gauss–Laguerre polynomials leads to an improved detection rate. As a result, detection of neoplastic lesions was achieved with an MCC of 0.47. Therefore, the classification accuracy of multispectral colonoscopy is comparable with hyperspectral colonoscopy in the same spectral range when additional spatial features are used. Moreover, this paper strongly supports the current path towards the application of multi/hyperspectral endoscopy in clinical settings and shows that the challenges from transferring results from ex vivo to in vivo endoscopy can be solved.

## Introduction

In Germany in 2014, more than 25% of the deaths were caused by cancer^1^. Even in 2020, cancer accounts for 23.5% of the deaths despite the pandemic situation^2^. From these, more than 25% were caused by cancer in the gastro-intestinal tract (GI)^1, 2^. An important reason for this high death toll of carcinomas in the GI tract lies in the difficulty of their detection. The main issue is that patients normally have symptoms only in later stages of the tumour development. Thus, the best chance to detect early stage carcinomas is regular screening by endoscopy. Currently, the state of the art is using thorough inspection with white light endoscopy with targeted biopsies, or random biopsies in inflammatory conditions. However, intraepithelial neoplasias, adenomas, and carcinomas are difficult to detect during screening colonoscopy, especially if they are flat and/or small in their macroscopic appearance^3–5^ or surrounded by inflamed tissue.

To overcome this problem, an approach centred around multi- and hyperspectral imaging (MSI, HSI) is proposed. MSI is often defined as having ten or less wavelength bands, while HSI is defined as having more than ten wavelength bands. Both MSI and HSI are increasingly used in medical applications^6, 7^. MSI and HSI combine machine vision with spectroscopy^8, 9^. They enable the acquisition of two-dimensional images with the spectral information for each pixel. Considerable progress has been made in many application fields^10^. So far, HSI has already been proven to be a reliable method for many medical applications^6^, including carcinoma detection in the oesophagus^11, 12^. However, most of the studies for endoscopic application have been performed ex vivo^8, 13, 14^. Moreover, there is currently no in vivo study employing MSI/HSI during colonoscopy known to the authors.

As discussed in a review by Swager and colleagues^15^ about gastrointestinal endoscopy, there is a need for further evaluation of spectroscopic quantitative measurements of tissue to allow a direct optical diagnosis of neoplastic lesions during endoscopy. It was already outlined before that such spectroscopic quantitative measurement can be provided by MSI and HSI. For MSI, the benefit could already be demonstrated by our group for the diagnosis of carcinomas of the stomach with an accuracy of 64%^16^ or an AUC of 0.72^17^ in vivo in patients. In this current in vivo study, MSI is applied in murine models of spontaneous and inflammation-driven carcinogenesis.

## Material and methods

### Set-up

**Figure 1:**
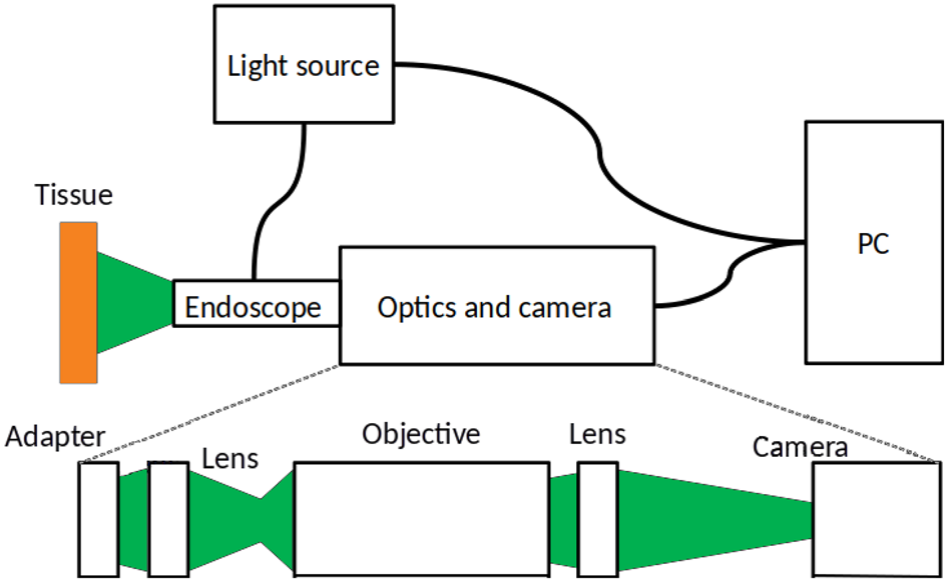
Schematics of the set-up (modified as described in our previous study^18^). The top schematic shows the overall set-up and the bottom schematic shows the optical set-up which images the light from the endoscope.

The set-up is a modified version from a Coloview high-resolution mouse endoscopy system (KARL STORZ SE & Co. KG, Germany). The mouse endoscopy system is a rigid endoscope which uses multiple lenses for imaging. Hence, there are no artefacts which occur as it happens from fibre bundles. This system was used with a spectral filter as a fluorescence endoscope system for the investigation of the topical application of Chlorin e6-PVP for improved endoscopic detection of neoplastic lesions^18^. The set-up is shown in Fig. 1.

The overall set-ups consists of the endoscope head (KARL STORZ SE & Co. KG, Germany) connected to a multispectral light source (Lumencor spectra 7-LCR-XA, Beaverton, OR, USA) and a custom optical system. Both systems are controlled by a personal computer (PC) via a graphical user interface (GUI). The GUI is written in Matlab (The MathWorks, Inc., Narick, MA, USA) and controls the data input, the data output and the external light source. The light source allows seven wavelength bands shown in Table 1. White light imaging is performed by activating all wavelength bands at the same time.

**Table 1:** Centre wavelength for the MSI device.

Wavelength in nm:	396	438	475	512	542	575	628
Colour	UV	Blue	Cyan	Teal	Green	Yellow	Red

The modified endoscope was already described in our previous publication^18^: “The custom optics unit incorporates two achromatic lenses with an apochromatic objective in between. The signal initially travels from the endoscopy telescope unit via an adaptor to the first lens with a focal length of 35 mm. This lens then conveys the real image to a 10$\times$ infinity corrected plan apochromat objective (Mitutoyo, Japan). The objective has a numerical aperture (NA) of 0.28 and a working distance of 34 mm. It images the received data to infinity. Using a 100 mm achromatic second lens, the image is finally transmitted from infinity to the camera unit (Basler ace ac2000-165ac USB3 color, Basler AG).” Out of the full resolution of 1280 $\times$ 1024 pixels, 520 $\times$ 496 pixels are acquired to speed up the imaging process of the whole multispectral image below 0.5 s. The boost of the sensor is set to 50% while the gain boost is turned off. In total, the optical set-up shows only small distortions. A small, nearly neglectable barrel distortion is present. Moreover, there is a small chromatic aberration at the outer parts of the image. The typical imaging errors of MSI and especially HSI devices (smile and keystone) are not present in this set-up. Due to the low amount of imaging errors, no image correction is performed.

### Mouse models and endoscopy procedure

*Ethical guidelines* Animal studies are approved by the Institutional Animal Care and Use Committee of the Friedrich-Alexander-Universität Erlangen-Nürnberg (FAU) (AZ 55.2-2532-2-365, AZ 54-2532.1-12/14) and the government of Lower Franconia. All methods were performed in accordance with the relevant guidelines and regulations. The study was carried out in compliance with the ARRIVE guidelines.

*Mouse models* In general, two models were used: inflammation-based carcinogenesis and spontaneous tumour models. The AOM/DSS model was employed to induce inflammatory carcinogenesis in 11 mice of C57BL/6 background as described previously^19^. The spontaneous model was based on the genetic APCmin or TP53 tumour model. The use of both models allowed to look for differences in the detection rate in neoplastic lesions of different disease mechanism.

*Endoscopy procedure* The endoscopy procedure is performed as described previously^20, 21^. In brief, isoflurane is used to establish anesthesia. Then, the bowel is carefully flushed with tap water to remove stool. Afterwards, the bowel is distended by means of careful air insufflation via the working channel. The colon is investigated with white light imaging and lesions that are deemed “definitive” neoplasms based on the assessment of their macroscopic appearance by the experienced endoscopists, were additionally evaluated with MSI. A biopsy for neoplasm assessment could not be done, as the mice were required for other purposes later on.

In total, 25 mice were investigated by this procedure. Each mouse had at least one neoplastic lesion. Lesions for spectral analysis were identified by two medical experts by classification as a “definitively neoplastic lesion” based on macroscopic appearance. To increase accuracy, assessment was performed by two medical experts. Only one of them drew the lesion margin.

For the MSI in this study, nine images were taken in the following order: one image for $\lambda =542$ nm, then the complete MSI series consisting of the seven wavelength bands as described in Table 1 and finally again one image for $\lambda =542$ nm. Before any further analysis, the first and the last image were compared. If both of them change significantly, the image was not used for further analysis due to movement-artefacts from the mouse. The margin of the lesion was drawn on the first and on the last image. Both margins were drawn in comparison with a video from a second endoscopic procedure of each mouse.

*Potential safety risks for human studies* The current imaging procedure imposes no major risks to potential patients compared to white light endoscopy as the required light intensities are similar. There are two potential minor risks: First, light with a wavelength of 397 nm is used. Long term exposure of blue light is known to increase the risk of skin carcinomas. However, the exposure time in this study is orders of magnitude lower than it is required for increased risks of carcinomas. Second, the usage of the MSI might increase the time of the endoscopic procedure in comparison to the white light endoscopic procedure.

### Data analysis

**Figure 2:**
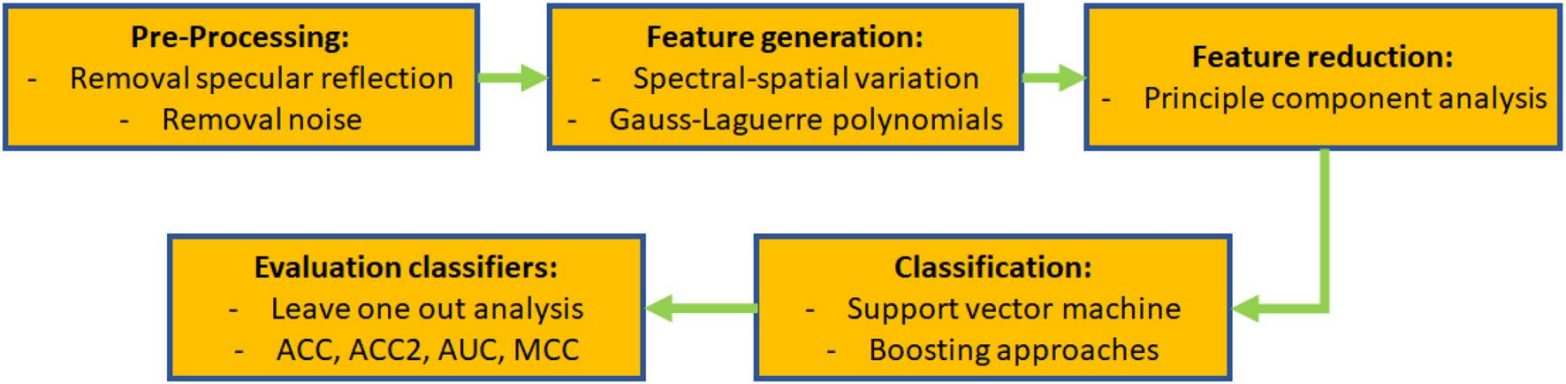
Flowchart of the data analysis. It groups in the five steps: pre-processing, feature generation, feature reduction, classification and evaluation of the classifiers.

The analysis is done with a pixel-per-pixel classification process. All data analysis is done with a leave-one-out strategy (LOOS) in which 24 mice are used for training and one for testing. No mouse was excluded from the analysis. In general, the data analysis is a modified version of that presented in two previous studies^16, 17^ with very detailed descriptions in the latter one^17^. In general, the data analysis consists out of the following steps shown in Fig. 2:

The classification process is described in detail later in this section. In the pre-processing step, noise is minimized and specular reflections are removed. Then, spatial and spectral-spatial features are generated. The number of features is reduced significantly by the principal component analysis (PCA) to use 99% of the variance in the next step. With the reduced feature set, the classification is done for support vector machine (SVM) and boosting classifiers. In the end, the results are evaluated.

*Pre-processing* The pre-processing consists out of four steps:

1. First, specular reflection and too dark areas are removed. If the intensity of a pixel in the red or yellow region is less than 5% of the maximal intensity, the pixel is excluded. Moreover, the surrounding with a distance of five pixels is also excluded. In total, about 1% of the data is excluded this way. Afterwards, Gaussian filtering is done with a filter size of seven by seven pixels with a standard deviation of one pixel to ensure that the minimum noise fraction (MNF) does not transmit some noise-caused artefacts from one wavelength band to others. As a last step, at the boundary between healthy tissue and neoplastic lesion a margin of five pixels is excluded due to the fact that there is a definite error in finding the tumour margin. In the next step, the data is normalized for every image separately.
  1 $$I_{norm}(x,y,\lambda ) = \frac{I(x,y,\lambda )}{ \displaystyle \max _{x,y} I(x,y,\lambda )}$$
  In Eq. 1, $I(x,y,\lambda )$ is the intensity of the multispectral image as the function of position and wavelength, $\max _{x,y}$ describes the function to take the maximum over each x and y and $I_{norm}(x,y,\lambda )$ is the normalized multispectral image. The normalization values ($\max _{x,y} I(x,y,\lambda )$) are saved for the de-normalization.
2. In this step, MNF^22^ is used for de-noising and enhancement of spatial features. Spectral bands with lower maximal intensity show a weaker influence while calculation of the MNF. Therefore, they were normalized before. In this study, the modified method from Regeling et al.^23^ is used. They altered it according to Gao et al.^24^. Furthermore, the MNF is not calculated by multi linear regression^22, 23^. It is changed to a regressive support vector machine (rSVM) as this significantly reduced artefacts from the MNF. The sub-image size for the MNF is 20 times 20 pixels and the last three MNF components are removed for noise filtering.
3. Afterwards, the data is de-normalized again as shown in Eq. 2:
  2 $$I(x,y,\lambda ) = \max _{x,y} I(x,y,\lambda ) \cdot I_{norm}(x,y,\lambda )$$
  where $I(x,y,\lambda )$ are the stored values used in Eq. 1.
4. As final step for pre-processing, a second Gaussian filter is used for further noise reduction with a size of 11 times 11 pixels and a standard deviation of two pixels.

*Feature generation* In general, a high number of features increases the classification accuracy. However in many cases, the amount of memory or the computational power limits the number of features. The limits are especially high for HSI, as there are already a high amount of spectral features. As MSI has often considerably fewer features, spatial features can be added without restriction due to limited memory or computational power. Therefore, four kinds of features are used: First, the intensity of the local multispectral image is used as a feature. Second, the derivative is used as a feature by the convolution with the Sobel operators. Third, the spectral-spatial variation is derived as shown before^17^. Forth, the spatial features based on Gauss–Laguerre polynomials (LGs) with *l* and *p* smaller 3 are used in the same way as described in a previous publication^17^.

**Figure 3:**
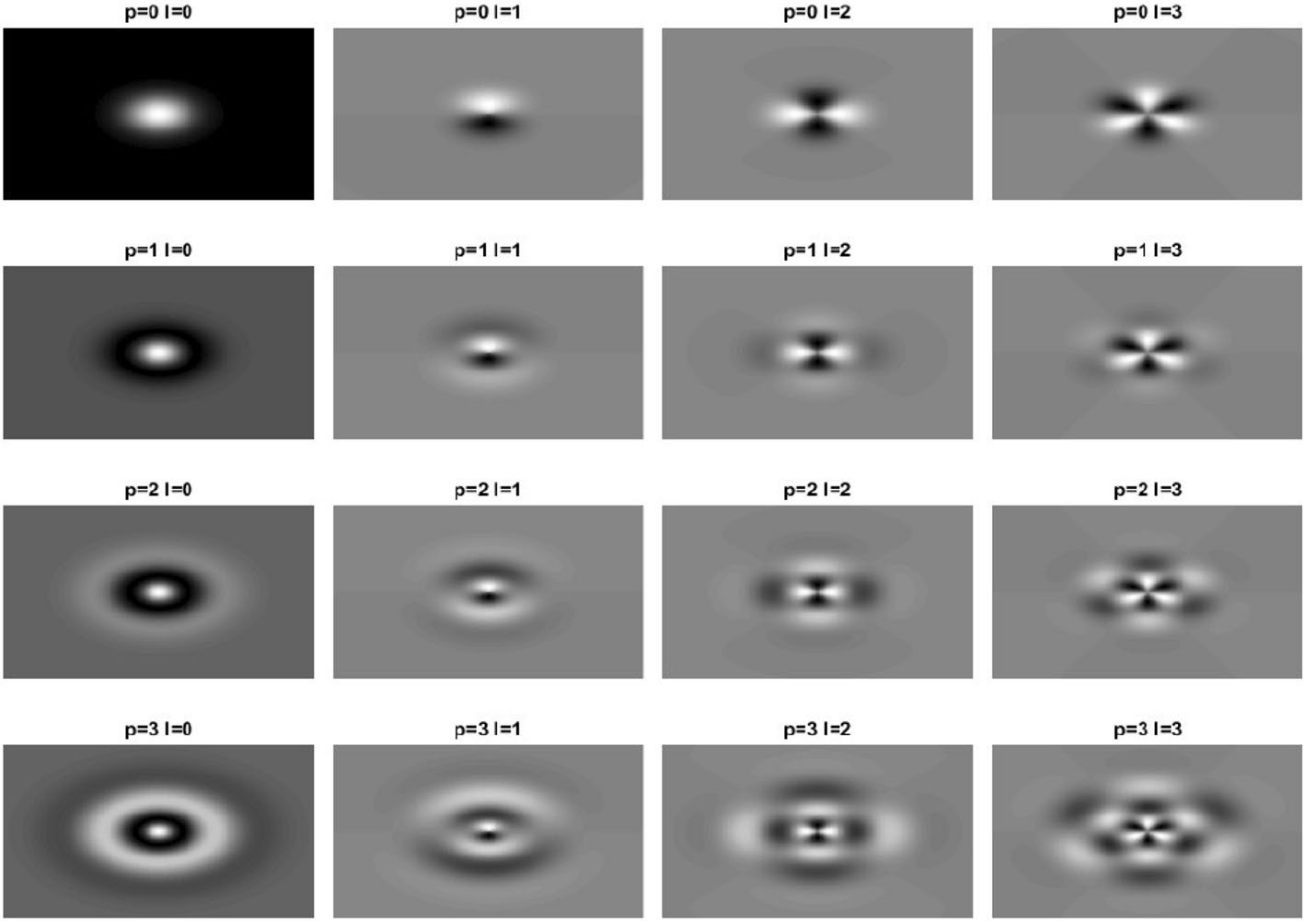
Example of the Laguerre–Gaussian polynomials.

The LGs are calculated as follows:

3 $$\begin{aligned} {}^r LG_l^p(x)& =\exp \left( (\rho \cdot s)^\frac{2}{q}\right) \cdot \left( \rho \cdot s\right) ^{\frac{l}{q}} \cdot \\ &\quad \left[ \cos \left( l \cdot \left[ \Theta + \frac{2r \pi }{\max (2l,1)}\right] \right) \cdot L_l^p\left( \rho \cdot s \cdot \arctan [\rho \cdot s)]\right) \right] ^{3-q} \end{aligned}$$

where *q* and *s* are constants, $\rho$ and $\Theta$ are the cylindrical coordinates and *r* is a parameter, describing image rotation with ($\{r \in \mathbb {R} \mid 0 \le r \le 1\}$). Thereby, *r* = 0 means no rotation and $r=1$ means the maximal rotation. The maximal rotation is half of the rotation which would be needed for an identity projection of the LGs. This is done due to the fact that $^0 LG_l^p(x)=- ^1 LG_l^p(x)$ and the fact that only the absolute value of the feature is used for further considerations. The constant q is set to 2 to generate amplitude images. In this study, *s* is set to 18, *p* is used from 0 to 3, *r* is varied from 0 to 1 in steps of 0.25 and *l* is used from 0 to 4. The constant *s* is selected so that the structure of the LGs is visible in a good way as shown in Fig. 3. The size of the LG features is 40 times 40 pixels.

Combined with the fact that just the absolute values are used, the amount of rotation can be halved with this ansatz and therefore speed up the calculation time. Moreover to measure the surrounding, only the highest absolute value is of importance due to the fact that different images might be rotated towards each other. Furthermore, one point of tissue under investigation might be imaged from different angles and, thus, only the feature with the best matching direction will be the one used as a feature. The best matching direction is found by selecting the highest value of the convolution for each set of *l* and *p* for all possible *r*. Therefore, the features generated with LGs are rotation invariant. This ansatz also reduced the number of features significantly and, thus, speeds up the analysis process.

*Feature reduction* While it is beneficial to have a large number of features, it is often the case that many features are redundant. There are many ways for feature reduction. However, generating combined features is often preferred as this also acts as noise reduction tool. For this, the PCA provides great results. In many cases, the results improve even in comparison to the usage of all features. Hence, the PCA is used for feature reduction. The features accounting for 99% of the variance are used for classification. This leads to approximately 30 features which are used for classification.

*Classification* As in the previous publications, RobustBoost (RB), AdaBoost (AB), SVM with linear and Gaussian kernel as well as random forest (RF) from the implemented Matlab functions are used for classification. The following commands are used: *fitensemble* for AB, RB; *fitcsvm* for SVM; *treebagger* for RF. Predictions were conducted using *predict* in all cases.

For RB, 100 stump trees are used with an error goal of 0.2. Replacing of the variables is not allowed, but resampling. Hence, it is the classical boosting approach with re-weighting the single trees. For AB, the learn rate is set to 0.1 and 400 stump trees are trained. Again, the classical boosting approach is chosen. For RF, 400 trees are trained and the minimal leaf size is set to 3. For both SVMs, standardization is used, the kernel scale is set to auto and outlier rate is set to 0.05. All other parameters are kept at standard settings.

To speed up the classification process, a random selection of 1% of the training data is used for training. It should be noted that this data reduction is done after the feature generation and reduction. Especially, the results would significantly degrade if the PCA would only be done on 1% of the data. The data points are selected randomly. For the test data, the whole data set is used. Furthermore, repetition of the classification process would not lead to significantly different classification results due to the random selection of the data points.

*Evaluation of the classifiers* For the evaluation of the classifiers, the four measures accuracy (ACC), modified accuracy (ACC2), area under the curve (AUC) and Matthews correlation coefficient (MCC) are used. They are used due to the fact that they are either well established in the medical field (ACC, AUC) or that they are balanced measures for the case that the amount of data from one class is much higher (ACC2, MCC). The ACC, ACC2 and MCC are defined as follows:

4 $$ACC= \frac{TP+TN}{TP+TN+FP+FN}$$

5 $$ACC2= \frac{Sen + Spe }{2}$$

6 $$MCC= \frac{TP \cdot TN - FP \cdot FN}{\sqrt{(TP+FP)(TP+FN)(TN+FP)(TN+FN)}}$$

where TP are the true positives, TN the true negatives, FP the false positives and FN the false negatives. The AUC describes the probability that a classifier will rank a randomly chosen positive instance higher than a randomly chosen negative one. The final accuracy measures are the mean of all 25 permutations from the LOOS. The data analysis is done with and without spatial features. Both are compared with an ANOVA. The ANOVA is done as threefold ANOVA with the following parameters: classifier, accuracy metric and usage of spatial features.

*Optimization of the parameters* There was only a slight optimization process done for this specific data set. Thereby, the following parameters were varied: sub-image size for the MNF (20 and 120) and amount of spatial features (P = L = 2 versus P = 3 and L = 4). The sub-image size for the MNF had nearly no effect while the increase of spatial features lead to a small improvement of the accuracy measures. The rest of parameters were chosen due to the fact that they showed good results of the classification of carcinoma tissue with MSI^17^. It is also not expected that wide parameter optimization would lead to much better results as the correct margin of the tumour is likely the reason which limits the quality of the results^25^.

## Results

The result section consists out of two parts. In the first section, a typical multispectral image is shown. In the second section, the results of the machine learning are shown and discussed.

### Example images

**Figure 4:**
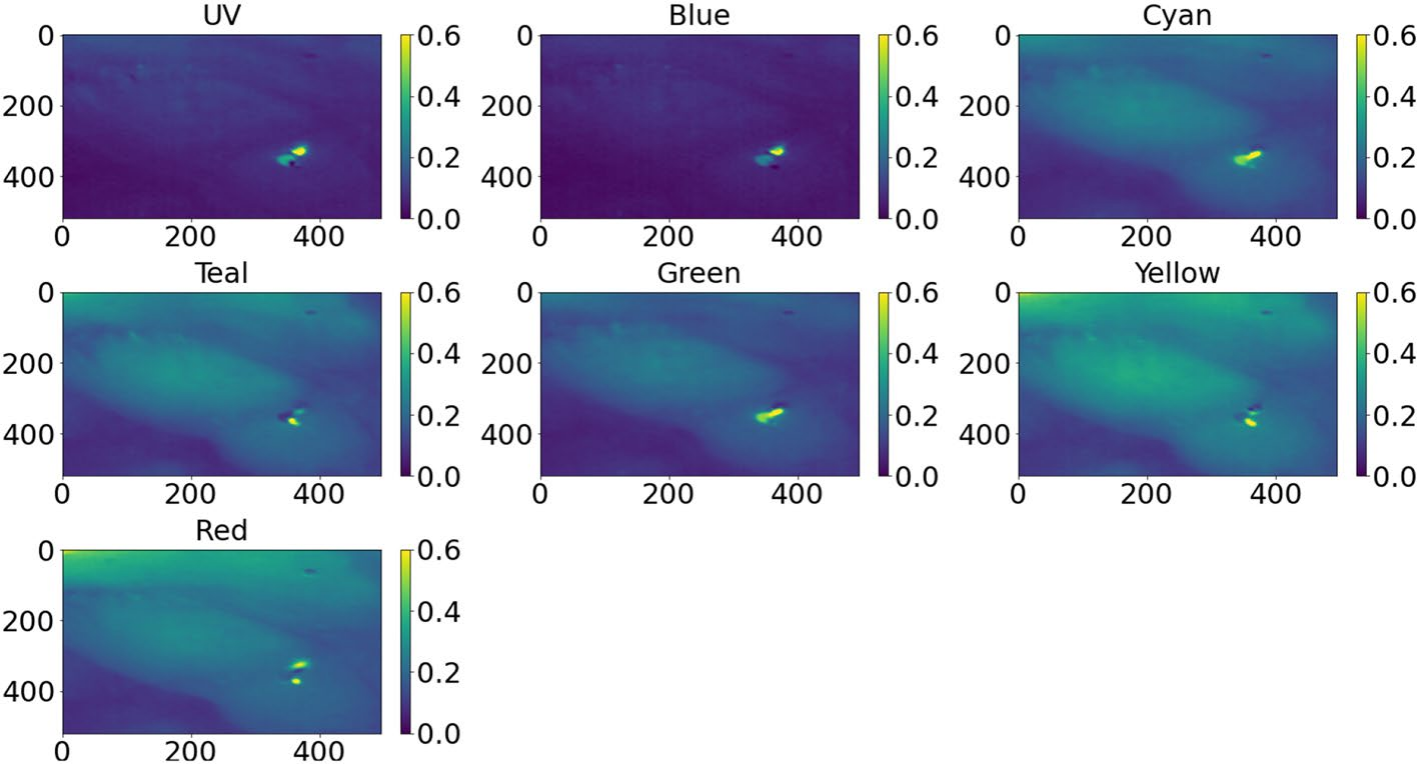
Example of multispectral image for the different wavelength bands. An example for the labelling of this multispectral image is shown in Fig. 2.

**Figure 5:**
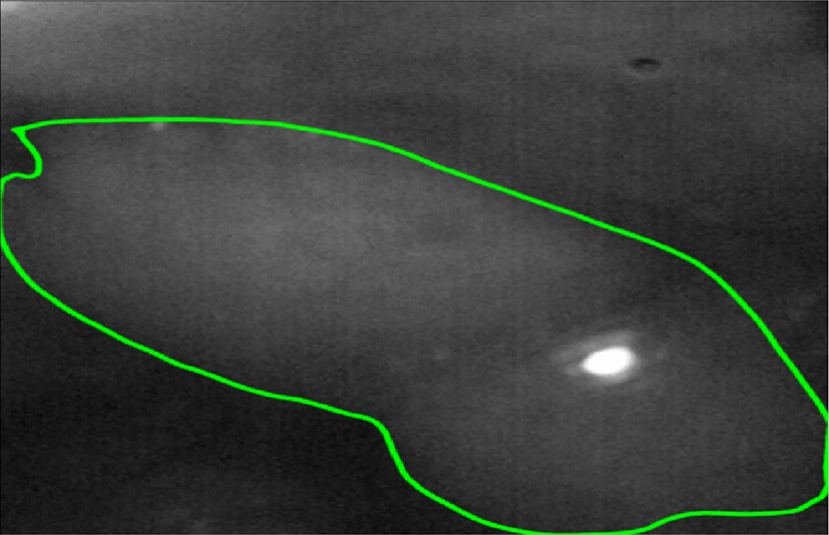
Example mouse image. The margin of the neoplastic lesion is marked in green.

Figure 4 shows a multispectral example image of a mouse. It is the same image as shown in Fig. 5. It can be seen that in the UV and blue range, the reflection is the lowest. Also the reflection in green is a little bit lower than in yellow or red. Moreover in the yellow and red range, the healthy tissue appears relatively brighter than the neoplastic lesion in the green range. Therefore, it is feasible to assume that the classification might work, as there are differences present. Furthermore, the specular reflection appears as two spots in Fig. 4. This happens due to the following two effects. First, the spectral scanning approach is used in this study. Hence between different wavelength bands, some movement can occur between different wavelength bands. Thus, the position of the specular reflection changes. By using the MNF-filtering, the specular reflection might appear also on other wavelengths.

### Classification

Table 2 shows the results for all five classifiers and all four accuracy metrics for the results with and without spatial features.

**Table 2:** Classification results of the tested classifiers for 25 mice with LOOS for the multispectral data set without (left side) and with spatial (right side) features.

Method	ACC	ACC2	AUC	MCC	ACC	ACC2	AUC	MCC
Data set	Without spatial features				With spatial features			
RF	0.67	0.64	0.73	0.39	0.72	0.73	0.76	0.46
RB	0.70	0.72	0.76	0.44	0.72	0.73	0.76	0.47
SVM (lin)	0.60	0.60	0.65	0.20	0.66	0.65	0.66	0.31
SVM (Gauss)	0.62	0.67	0.68	0.34	0.63	0.65	0.68	0.31
AB	0.68	0.70	0.75	0.41	0.73	0.73	0.76	0.47

The PCA is done and 99% of the variance of the PCA is used.

The results are strongly dependent on the chosen classifier as well as on the chosen accuracy metric. Nevertheless, there is an overall increase by the usage of spatial features especially for weaker classifiers such as the SVM with linear kernel. Nevertheless, even the results for the stronger classifiers such as RB, AB and RF improve a little bit. In general, the classification between healthy and neoplastic lesion in mice provides better results than the in vivo clinical study on oesophagus in humans in our previous studies^16, 17^. The best classifier is RB for all measures. Thus, it is likely that the training data has misclassified data. Therefore, it is likely that the margin of the neoplastic lesion was not correctly identified by the medical expert.

Table 3 shows the comparison to other groups. The overall results generated in this in vivo study reach a similar ACC, AUC and MCC as in the HSI study from Baltusen et al.^26^ in the visible range despite their study is an ex vivo study. However, similar results can only be reached for the MSI data set with spatial features. This pinpoints that the usage of spatial features is a very important step in future for multispectral endoscopy. Furthermore, the MCC in this study is nearly identical to the ex vivo results from Collins et al.^27^ in which they used a 3d convolutional neural network (3DCNN). Finally, the multispectral data set with spatial features will likely have a similar data dimensionality as hyperspectral data sets. Hence, it is a valid alternative.

**Table 3:** Comparison with the results from other groups.

Study/year	Spectral range (nm)	Ex vivo/in vivo	MCC	AUC	ACC
Baltusen et al.^26^/2019	400–1000	*Ex vivo*	0.50	0.81	0.74
Baltusen et al.^26^/2019	900–1600	*Ex vivo*	0.59	0.87	0.80
Baltusen et al.^26^/2019	400–1600	*Ex vivo*	0.83	0.98	0.91
Collins et al.^27^/2021	500–1000	*Ex vivo*	0.49	0.93	–
This study with spatial features	400–630	*In vivo*	0.47	0.76	0.73

An ANOVA shows significant differences ($\hbox {p}<0.0005$) for each of the following parameters: the kind of accuracy measure, the classifier and the spatial features. From these, the accuracy measure explains most of the variance ($\partial \eta ^2=0.53$). This is expected as the MCC has its values in a different range than e.g. the ACC. The classifier explains the second most variance ($\partial \eta ^2=0.07$). The features only explain around 1% of the variance ($\partial \eta ^2=0.01$). However despite the low $\partial \eta ^2$, the spatial features are still required to push the results in the region from the study from Baltusen et al.^26^.

Table 4 shows the different classification results for the inflammation driven tumour models as well as the spontaneous tumour models. The results with spatial features are presented in Table 4. Between both results, there is no significant difference in the final classification accuracy ($\hbox {p}>0.3$; ANOVA). Hence the typical issue that inflammation driven neoplastic lesions are more difficult to find does not seem to appear in this study.

**Table 4:** Classification results of the tested classifiers for the spontaneous cancer model (left) for 14 mice and the inflammation driven cancer model (right) for 11 mice.

Method	ACC	ACC2	AUC	MCC	ACC	ACC2	AUC	MCC
Cancer model	Spontaneous (n = 14)				Inflammation driven (n = 11)			
RFW	0.73	0.74	0.77	0.48	0.71	0.71	0.74	0.43
RB	0.72	0.74	0.77	0.48	0.72	0.72	0.76	0.45
SVM (lin)	0.65	0.64	0.65	0.30	0.68	0.66	0.68	0.32
SVM (Gauss)	0.62	0.63	0.66	0.27	0.64	0.67	0.70	0.36
AB	0.73	0.74	0.77	0.48	0.72	0.73	0.75	0.46

Table 5 shows the MCC as a function of spatial features and if inflammation driven tumour models are used or not. It can be seen that the improvement due to the usage of spatial features is similar for the inflammation driven and the spontaneous tumour model. Thus, both tumour models seem to have distinct alterations of the colon. Hence, there does not seem to be an effect of disguise caused by the inflammation. Nevertheless, the results from the different classifiers are contradicting. Hence, more analysis of this is required in the future.

**Table 5:** Classification results of the MCC for all five classifiers as a function of spatial features and if inflammation driven tumour models are used or not.

Spatial features	No	Yes	No	Yes
Cancer model	Spontaneous (n = 14)		Inflammation driven (n = 11)	
RFW	0.41	0.48	0.37	0.43
RB	0.43	0.48	0.45	0.45
SVM (lin)	0.24	0.30	0.15	0.32
SVM (Gauss)	0.36	0.27	0.32	0.36
AB	0.41	0.48	0.41	0.41

## Conclusion and summary

MSI with support of spatial features allowed classification of neoplastic lesions in the colon. Normally there is an expected advantage of real HSI over MSI, HSI has two major drawbacks. The first one is the curse of dimensionality. The problem is that an increase in dimensionality leads to a fast increase of the volume of the feature-space so that the available data become sparse, making classification problems much harder to solve. Second, the high correlation between continuous spectral bands^28^ must be considered. This leads to the problem that - in combination with the sparsity of the data - it is difficult to find the correlations between spectral bands. Due to these points, HSI will be very difficult to set up in future. A further minor issue is that the margin of carcinomas is not known for in vivo endoscopy^29^.

Despite the expected advantage of real HSI over MSI, the above points emphasize why it still seems to be an attractive option to revert to MSI. Additionally, this is an issue well known in remote sensing: Not all HSI spectral bands are required as some of them already include all significant information^30, 31^. Thus, it might make sense to switch back to MSI after the optimal spectral bands have been found by HSI; potentially, it might not even be required to use the HSI at all as almost all absorbers in tissue have a broad spectral effect. Finally, MSI set-ups are also easier to build.

This line of arguments is supported by the fact that the classification results of the present study are similar quality to the results from the ex vivo HSI study from Baltusen et al.^26^ in the visible range; however, their results are superior in the NIR range from 1000 to 1600 nm and their combination of NIR and VIS leads to further huge improvements. This allows the conclusion that multispectral endoscopy with a few additional wavelengths in the NIR region is likely to show the same promising results as full HSI without the downside of the latter.

The main limitation of the present study is the fact that there was a trade-off between the number of available mice and the possibility to perform biopsies as gold standard for the evaluation the neoplastic lesions. This trade-off was solved at the expense of omitting the biopsies, allowing to investigate 25 mice—a quite substantial number. Therefore, only neoplastic lesions without further differentiation could be used as target for classification.
